# Impact of lifestyle education for type 2 diabetes mellitus: Protocol for a randomized controlled trial

**DOI:** 10.1097/MD.0000000000024208

**Published:** 2021-01-08

**Authors:** Jingjing Zhu, Min Chen, Yuzi Pang, Shumin Li

**Affiliations:** Department of Endocrinology, Ningbo No.6 hospital, Zhejiang, China.

**Keywords:** lifestyle education, microalbuminuria, protocol, type 2 diabetes mellitus

## Abstract

**Objective:**

To explore the influence of the education of lifestyle in the type 2 diabetes mellitus (T2DM) patients with microalbuminuria as a part of the enhanced multifactorial intervention.

**Methods:**

This study will be conducted from May 2021 to August 2022 at Ningbo No.6 hospital. The experiment was granted through the Research Ethics Committee of Ningbo No.6 hospital (539D035). The patients will be included if they are between 18 and 65 years old and are diagnosed with T2DM with microalbuminuria and the patients who have signed the written informed consent. While the patients will be excluded if they have serious physical comorbidities and patients who are unwilling to offer the informed consent to take part in this experiment. We measure the clinical examination (fasting blood-glucose, glycosylated hemoglobin and routine urine test) timely. Detail of daily dietary intake and lifestyle factors are also recorded.

**Results:**

Table 1 reflects the comparison of the biochemical and clinical variables and the lifestyle factors.

**Conclusion:**

Lifestyle education is effective in facilitating the control of T2DM and reducing microalbuminuria.

**Trial registration number:**

researchregistry6348.

## 1 Introduction

Type 2 diabetes mellitus (T2DM) is regarded to be one of the most prevalent diseases worldwide.^[1,2]^ For the T2DM, its etiology is complex, which is related to the reversible factors (for instance, smoking, physical activity and diet) and the irreversible risk factors (for instance, genetics, age, ethnicity and race).^[3,4]^ Due to the urbanization, the aging of the population, and the changes of lifestyle, the prevalence of diabetes mellitus is rising rapidly in the world. In the past 30 years, the number of diabetes mellitus people in the world has more than doubled.^[5]^ In 2010, approximately 285 million people suffered from diabetes mellitus in the world, 90 percent of these patients were T2DM. It is estimated that by 2030, the number of diabetes mellitus patients worldwide will increase to 439 million.^[6]^ Diabetic nephropathy is one of the most feared and prevalent complications of T2DM.^[7]^ With the increasing burden of diabetes in the world, an increasing number of diabetic nephropathy patients have emerged. It is also related to the increased mortality rate, and is one of the most familiar causes of renal replacement therapy and end-stage renal disease. Mental stress, inadequate physical activity and improper diet are regarded as the main components of the unhealthy lifestyle, which are the significant factors leading to the onset of T2DM.^[8–10]^ Recently, more and more studies have emphasized the value of lifestyle education in patients with T2DM ^[11,12]^ and the certificated diabetes educator is considered an important member of the multidisciplinary team responsible for patient care. Education of lifestyle for patients with T2DM is primarily carried out in hospitals. Nevertheless, offering education in the outpatient department should effectively improve the participation of T2DM patients in the education of lifestyle. Currently, the efficacy of lifestyle education in T2DM with microalbuminuria has not been consistently demonstrated. Therefore, the purpose of this protocol is to explore the influence of the education of lifestyle in the T2DM patients with microalbuminuria as a part of the enhanced multifactorial intervention.

## 2 Methods

This study will be conducted from May 2021 to August 2022 at Ningbo No.6 hospital. The experiment was granted through the Research Ethics Committee of Ningbo No.6 hospital (539D035) and recorded in research registry (researchregistry6348). The random numbers sequence is produced through the computer. The random numbers are hidden by the sequentially numbered opaque and sealed envelopes. The patients will be randomly assigned into the study group and control group and each group includes 50 patients. The duration of follow up is 2 months.

### 2.1 Inclusion and exclusion criteria

Patients will be included in this experiment if their ages are in the range of 18 to 65 years old and are diagnosed with T2DM with microalbuminuria and the patients who have signed the written informed consent. While the patients will be excluded if they have serious physical comorbidities and patients who are unwilling to offer the informed consent to take part in this experiment.

### 2.2 General education

A detailed explanation of the pathogenesis of diabetes, use of medications and the effect of self-monitoring devices are provided to address patients concerns and queries. The family members of patients are encouraged to help monitor the medication compliance.

### 2.3 Diet

Prior to randomization, all patients are informed of the fundamental principles of diabetes diet. The goal of the study group is to achieve permanent changes in the quantitative and qualitative dietary fat by a low intake of saturated fatty acids, the high intake of monounsaturated fatty acids and polyunsaturated fatty acids and increasing the intake of the complex carbohydrates. The whole team provides a large number of personal dietary consultations. Consultations are the personal interviews. The patients risk profile is discussed and the individual goals for smoking cessation, exercise, and diet are set and modified in collaboration with patient. The topics include smoking, exercise and diet habits, combined with the discussions of lifestyle habits, beliefs and attitudes. The method of education is on the basis of the patients needs.

### 2.4 Exercise

Exercise refers to the physical activities other than daily chores and work activities. Through the educational exercises and positive feedback, for instance, proving that moderate physical exercise can immediately reduce blood glucose, thus prompting patients to start or continue to exercise.

### 2.5 Smoking habits

All the smokers and their spouses in study group are invited to participate in the programs of standardized smoking cessation. The project is carried out in the form of group consultations with a maximum of 14 participants. Patients are educated about the benefits of quitting smoking, and the specific advice on how to cope with the temptation to start smoking again, containing the information on the substitutes of nicotine, which is provided free of charge.

### 2.6 Outcomes

We measure the clinical examination (fasting blood-glucose, glycosylated hemoglobin and routine urine test) timely. Detail of daily dietary intake and lifestyle factors are also recorded.

### 2.7 Statistical analysis

Through utilizing the Microsoft Excel 2013, the data is recorded, and the analysis of all the data is carried out through utilizing IBM SPSS Statistics for Windows, version 20 (IBM Corp., Armonk, NY, USA). With the χ^2^-tests and independent *t* tests, the analysis for continuous variables and categorical variables are implemented respectively. All data are described with proper features, such as percentage, average and median. *P* value less than .05 indicates that there is statistical significance.

## 3 Results

Table 1 reflects the comparison of the biochemical and clinical variables and the lifestyle factors.

**Table 1:** Comparison of the biochemical and clinical variables and the lifestyle factors.

	Study group (n = 50)	Control group (n = 50)	*P* value
Body weight			
Current smokers			
Glycosylated hemoglobin			
Fasting blood-glucose			
Energy intake			

### 3.1 Discussion

The number of T2DM patients is increasing rapidly, which has become a serious problem of health in the world. Obesity and sedentary lifestyle have been linked to an increase in the T2DM prevalence globally.^[13,14]^ For a long time, the educators of diabetic nursing have always regarded lifestyle education as the cornerstone of diabetes teaching, aiming to control the concentrations of blood glucose, help the patients improve their medical compliance, and slow down the progress of disease-related complications.^[15,16]^ The management of T2DM involves exercise, diet, and medication.^[17,18]^ However, adherence to these lifestyle measures has been sub-optimal in usual care for diabetes, leading to higher rates of mortality related to diabetes and poor glycemic control. In order to improve the prognosis of T2DM, we design the programs of structured lifestyle intervention. Considering that the sample size of our study is small and follow up is short, further high quality randomized controlled trial is required.

## 4 Conclusion

Lifestyle education is effective in facilitating the control of T2DM and reducing microalbuminuria.

## Author contributions

**Conceptualization:** Min Chen.

**Data curation:** Min Chen.

**Funding acquisition:** Shumin Li.

**Investigation:** Yuzi Pang.

**Methodology:** Yuzi Pang.

**Writing – original draft:** Jingjing Zhu.
